# Supplementary material for: In vitro 3D drug sensitivity testing for patient-derived tumor-like cell clusters and whole-exome sequencing to personalize postoperative treatment: A study protocol for a multicenter randomized controlled trial
Source: PLoS One. 2025 Jul 14;20(7):e0326760. doi: 10.1371/journal.pone.0326760 (PMC12258565; doi:10.1371/journal.pone.0326760)
Supplement: S1 File — (PDF) [file pone.0326760.s001.pdf]

Supplement to Manuscript Submission for PLoS One

Protocol

In vitro 3D drug sensitivity testing for patient-derived tumor-like cell clusters and whole-exome sequencing to personalize postoperative treatment

Version: 1.0 Date 2021-9-28

PI：Guole Lin

Project undertaking unit: Peking Union Medical College Hospital

Abstract

**Purpose**
To compare the results of micro-tumor (PTC) ex vivo 3D drug sensitivity testing with the clinical outcomes

of patients, to evaluate the consistency between the test results of this technology platform and clinical prognosis, and to explore the decision-making value and guiding significance of this technology for assisting in the precision treatment of colorectal cancer. The completion of this study will provide real-world data support for the application of micro-tumor (PTC) ex vivo 3D drug sensitivity testing in clinical practice, and offer more valuable reference basis for achieving personalized and precise treatment of colorectal cancer patients, and improving the clinical benefit rate.	
**Design**	
Research Type	Prospective
Observation Point Type	Cohort Study
Scope of Study	Domestic Research
**Study Timeline**	
Enrollment Start Date	September 2021
Study Duration	3 years
**Research Participants**	
Age	18~75
Gender	Male + Female
Healthy Participants	No
Inclusion criteria	1) patients with histologically confirmed CRC;<br>2) baseline clinical stage of cT3-4N0M0 or cTanyN+M0;<br>3) CRC patients requiring adjuvant treatment after radical surgery and not receiving neoadjuvant treatment;<br>4) at least one evaluable tumor lesion;<br>5) Eastern Cooperative Oncology Group (ECOG) performance status ≤ 2.
Exclusion criteria	1) patients with distant metastases;<br>2) patients unable to obtain tumor samples;

	3) pregnant and lactating women;<br>4) patients with poor compliance;<br>5) patients with severe cardiovascular and cerebrovascular complications unable to receive chemotherapy or targeted therapy;<br>6) patients previously diagnosed with other malignant tumors;<br>7) patients with severe mental and neurological disorders;<br>8) patients deemed unsuitable for participation in this study by researchers.
Withdrawal	1) Subjects may voluntarily withdraw from the trial at any time.<br>2) If the investigator deems that continuing participation in the clinical trial would be harmful to the subject's health.<br>3) According to the specific requirements of the sponsor.<br>4) Subject is lost to follow-up.<br>5) Non-compliance with study requirements, serious protocol deviation.<br>6) Use of medication or other substances may induce toxicity or cause bias in the results.<br>7) Concurrent diseases or conditions may occur, potentially affecting the assessment of clinical status and study endpoints.<br>8) Pregnancy or suspected pregnancy.<br>9) Other circumstances occurring during the trial process that may impact the endpoint results.
**Research Grouping and Intervention Measures**	
PTC Drug Sensitivity Test Group:	Selecting adjuvant chemotherapy regimen based on the results of micro-tumor (PTC) ex vivo 3D drug sensitivity testing.<br>Collecting whole exome sequencing (WES) data to predict postoperative survival.
Control Group:	Formulating adjuvant chemotherapy strategy based on clinical experience.<br>Collecting PTC drug sensitivity and WES data.<br>The study is planned to be completed within 3 years. Once positive results are

	obtained, the data can subsequently be used to guide treatment.
**Endpoints**	
Main endpoint	3-year disease-free survival (3yDFS) rate
Secondary endpoints	1) consistency between the test results of the extended medication regimen and the clinical outcomes;<br>2) time to progression (TTP), objective response rate (ORR), disease-free survival (DFS), progression-free survival (PFS), and overall survival (OS);<br>3) consistency of gene mutation status, tumor mutation burden (TMB), microsatellite instability (MSI), and defective mismatch repair (dMMR) status with clinical prognosis.

1 BACKGROUND

1.1 Colorectal cancer

Colorectal cancer is one of the common malignant tumors of the digestive tract that severely endangers human health. In China, there are more than 500,000 new cases and nearly 300,000 deaths each year, putting tremendous pressure on the health care system. Adjuvant chemotherapy, as the main adjuvant treatment for colorectal cancer surgery, has made significant progress over the past decade, and numerous international clinical studies have confirmed the role of adjuvant chemotherapy in the postoperative treatment of colorectal cancer.

After surgery alone, about 25% of stage II colorectal cancer patients will experience recurrence and metastasis within 5 years, and about 50-60% of stage III colorectal cancer patients will experience recurrence and metastasis within 5 years. Multiple international clinical study results show that surgery combined with adjuvant chemotherapy can achieve a total 5-year survival rate of 60% to 70% for all stage II and III patients, and a total 5-year disease-free survival rate of 55% to 65%.

However, colorectal cancer is a malignant tumor with significant heterogeneity, and patients exhibit significant individual differences in drug sensitivity. Patients with the same pathological type and clinical stage may have different prognoses and outcomes even with the same treatment plan. There are still different views at home and abroad on the timing of adjuvant chemotherapy and the choice of chemotherapy regimens for different subgroups of patients. Therefore, constructing a model that can accurately predict drug effects to guide individualized treatment for patients is one of the strategies to achieve effective treatment of colorectal cancer.

1.2 In vitro 3D drug sensitivity testing for patient-derived tumor-like cell clusters

Recently, the research group led by Professor Xi Jianzhong from the School of Engineering at Peking University, in collaboration with teams from Peking University Cancer Hospital, Peking University People's Hospital, and Beijing Cornerstone Life Science Co., Ltd., has jointly developed a brand-new ex vivo model based on patient-derived primary tumor cells, known as Patient-Derived Tumor-like Cell Clusters (PTC, micro-tumor PTC model). They have established a tumor ex vivo drug sensitivity testing technology based on PTC. The research findings were published in the journal Science Translational Medicine under the title "Patient-Derived Tumor-like Cell Clusters for Drug Testing in Cancer Therapy" with an impact factor of 17.2.

The micro-tumor PTC model is a 3D microsphere formed by the self-assembly of tumor cells and tumor stromal cells derived from patient samples after cutting, digestion, and proliferation. It has been validated to have a high degree of similarity with the original tumor tissue in terms of morphological structure, pathological characteristics, genetic background, and other dimensions (Figure 1).

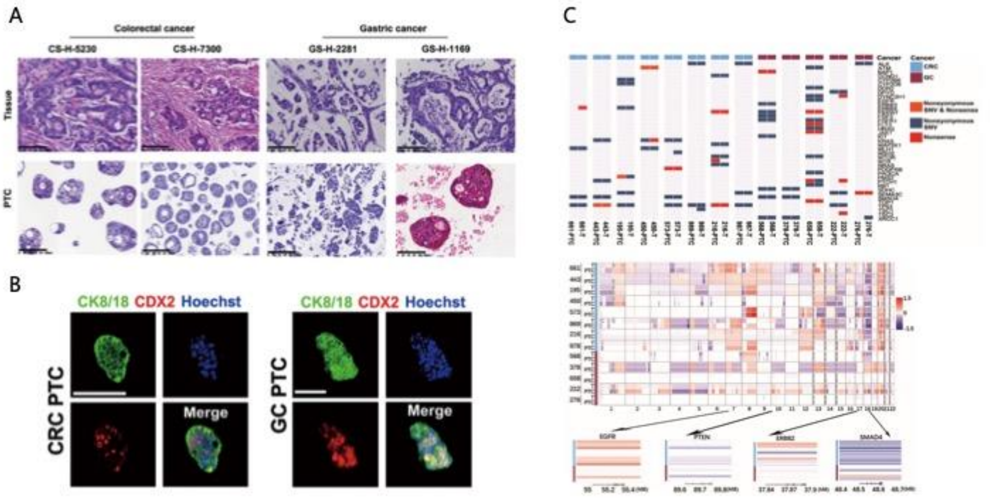

Figure 1. Consistency between PTC Model and Tumor Tissue

The PTC model comprises a variety of cells derived from tumor tissue, including tumor epithelial cells, tumor stem-like cells, fibroblasts, immune cells, etc., which can simulate the tumor microenvironment to a certain extent (Figure 2).

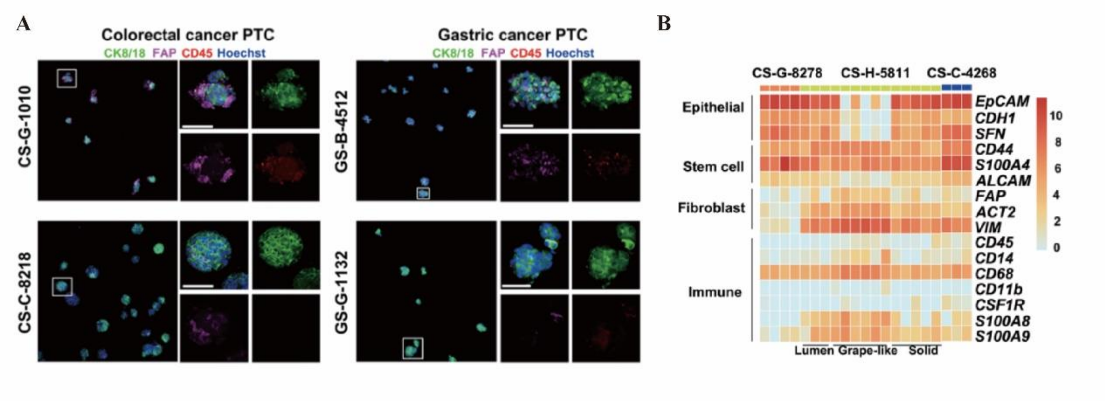

Figure 2. Analysis of Cellular Components in the PTC Model

A clinical double-blind validation using the micro-tumor PTC drug sensitivity testing model was conducted on 24 gastrointestinal tumor patients who were enrolled and had PTC models available. The clinical efficacy was assessed, and the consistency between the PTC drug sensitivity test results and clinical efficacy in gastrointestinal tumors reached 96.6% (Figure 3). This preliminary result confirms the high accuracy of the PTC drug sensitivity testing technology. These findings provide a foundation for the successful implementation of the project.

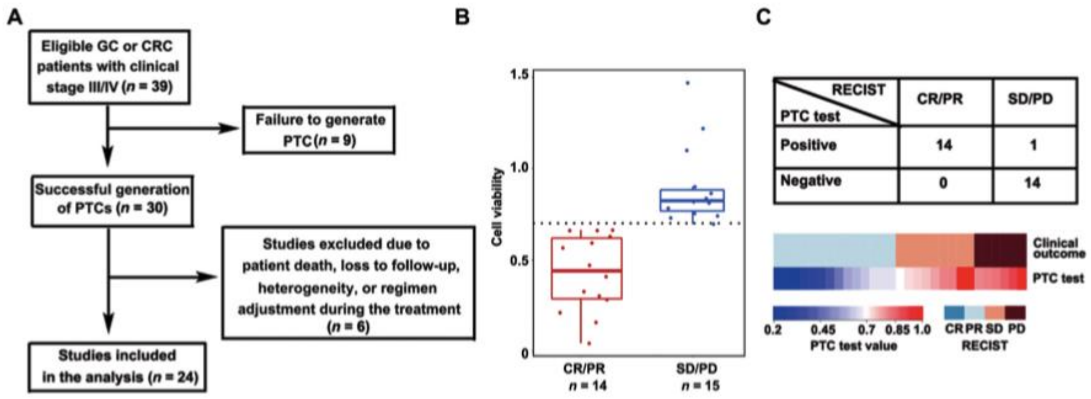

Figure 3. Consistency between PTC Drug Sensitivity Test Results and Clinical Efficacy in Gastrointestinal Tumors

2 PURPOSE

Comparing the results of the micro-tumor (PTC) ex vivo 3D drug sensitivity test with the clinical outcomes of patients, evaluating the consistency between the test results of this technology platform and clinical prognosis, and exploring the decision-making value and guiding significance of this technology for assisting in the precision treatment of colorectal cancer. The completion of this study

will provide real-world data support for the clinical application of the micro-tumor (PTC) ex vivo 3D drug sensitivity testing technology, and provide more valuable reference basis for achieving personalized and precise treatment for colorectal cancer patients, and improving the clinical benefit rate.

3 DESIGN

Prospective, multicenter study, planning to enroll 200 patients in this research.

4 CANDIDATES

4.1 Inclusion Criteria

1) patients with histologically confirmed CRC;

2) baseline clinical stage of cT3-4N0M0 or cTanyN+M0;

3) CRC patients requiring adjuvant treatment after radical surgery and not receiving neoadjuvant treatment;

4) at least one evaluable tumor lesion;

5) Eastern Cooperative Oncology Group (ECOG) performance status ≤ 2.

4.2 Exclusion Criteria

1) patients with distant metastases;

2) patients unable to obtain tumor samples;

3) pregnant and lactating women;

4) patients with poor compliance;

5) patients with severe cardiovascular and cerebrovascular complications unable to receive chemotherapy or targeted therapy;

6) patients previously diagnosed with other malignant tumors;

7) patients with severe mental and neurological disorders;

8) patients deemed unsuitable for participation in this study by researchers.

4.3 Withdrawal Criteria

1) Subjects may voluntarily withdraw from the trial at any time.

2) If the investigator deems that continuing participation in the clinical trial would be harmful to the subject's health.

3) According to the specific requirements of the sponsor.

4) Subject is lost to follow-up.

5) Non-compliance with study requirements, serious protocol deviation.

6) Use of medication or other substances may induce toxicity or cause bias in the results.

7) Concurrent diseases or conditions may occur, potentially affecting the assessment of clinical status and study endpoints.

8) Pregnancy or suspected pregnancy.

9) Other circumstances occurring during the trial process that may impact the endpoint results.

5 DETAILED IMPLEMENTATION PLAN

5.1 Patient Enrollment and Sample Collection for PTC Model Construction

5.1.1 Patient Recruitment

Patients are recruited based on the normal clinical requirement for biopsy or surgery. Before the procedure, researchers assess whether the patients meet the eligibility criteria. Eligible patients are provided with a paper Informed Consent Form (ICF), which is explained in clear and colloquial terms. The explanation includes the purpose, content, process, potential risks and benefits, rights, and obligations of the study, and answers any questions the patient may have during recruitment. If the patient agrees to participate, they sign the ICF to confirm their consent. After signing the ICF, the patient undergoes further assessment to determine if they meet the study criteria.

5.1.2 Sample Collection

5.1.2.1 Surgical Specimen Acquisition:

Sample collection tubes are provided by the leading unit before surgery. Tubes are 15 mL sterile centrifuge tubes containing 5 mL of sample preservation fluid, stored at 4°C with a 6-month shelf life.

The sampling environment is within the operating room after tumor resection to avoid microbial contamination. Select fresh cancerous tissue rich in blood vessels, avoiding necrotic areas, fibrous regions, fatty tissue, and tissue that has been cauterized by an electric scalpel, to preserve cell viability in the tissue. A minimum of 50 mg of tumor tissue sample is required.

After collection, the sample is immediately stored in a 4°C refrigerator and the leading unit is notified for sample collection and logistics arrangement.

5.1.2.2 Biopsy Specimen Acquisition:

Collection tubes are provided by the leading unit before the procedure. Tubes are 2 mL sterile cryopreservation tubes containing 1 mL of sample preservation fluid, stored at 4°C with a 6-month shelf life. The biopsy specimen should be immediately placed in the preservation tube without contact with filter paper.

The sample quantity should be 3-5 endoscopic specimens or 3-5 needle biopsy specimens. After collection, the sample tube is immediately stored in a 4°C refrigerator and the sampling personnel are notified for sample collection and logistics arrangement.

5.1.3 Sample Management and PTC Model Construction

Samples are temporarily stored at 4°C and sent to the central laboratory for processing within 24 hours. The laboratory checks the sample number and creates a sample file in the laboratory management system for data file management. After inspecting the sample status (including temperature, time out of the body, and any leakage), the sample is processed and cultured.

Cell culture is observed under a bright-field microscope for 2-7 days to monitor the formation of PTC clones.

If no PTC clones are formed within 7 days, the culture is declared a failure, and the patient is withdrawn from the study. If PTC clones are formed, a bright-field photo is taken, and the culture is tested for bacterial, fungal, and mycoplasma infections. Samples that test negative for infections are eligible for drug sensitivity testing.

5.1.3.1 PTC Drug Sensitivity Testing

For the PTC drug sensitivity testing, a list of drugs is used as indicated in Table 1. The criteria for determining the results of the micro-tumor PTC drug sensitivity test are outlined in Table 2.

5.1.3.2 Drug Screening

The screening experiment uses a 96-well plate. Each experimental group has three parallel replicate wells to ensure the reliability of the experimental data.

5.1.4 Drug Sensitivity Result Interpretation

Each sample undergoes two data collections: one at day 0 (before drug addition) and one at day 7 (after drug addition).

Data collection is performed automatically using a scanning system that identifies the number and area of all PTC clones in each well.

The area ratio of PTC clones after drug addition to before drug addition (i.e., the remaining cell

viability) is calculated using the following formula: $p_{Ai} = S_{Ai,t1}/S_{Ai,t0}, p_A = \frac{1}{n}\sum_{i=1}^{n} p_{Ai}$. The interpretation criteria are as follows (Table 2).

Table 1: Colorectal Cancer PTC Drug Sensitivity Test List

Drug ID	Drug Combination
001	5-Fluorouracil + Leucovorin
002	Oxaliplatin + 5-Fluorouracil + Leucovorin
003	Irinotecan + 5-Fluorouracil + Leucovorin
004	Cetuximab + 5-Fluorouracil + Leucovorin

Table 2: PTC Drug Sensitivity Test Result Interpretation Criteria

Killing Grade	Killing Effect	Remaining Cell Viability	Notes
Strong Killing	>0.7	0-0.3	
Effective Killing	0.3-0.7	0.3-0.7	If several plans fall within this range, for example, both Plan A and Plan B have killing effects within this range, choose Plan A if A-B is >=10%, choose the plan with less toxicity if A-B is <10%
Stable	0.1-0.3	0.7-0.9	
Drug Resistance	0-0.1	0.9-1	
Strong Drug Resistance	0	1	

5.2 Patient Standardized Treatment and Follow-up Data Tracking

Patients undergo standardized treatment, and follow-up data tracking is conducted to assess the following: Record of Survival Status, Progressive Tumor Assessment via CT or MRI Scans, Blood Tumor Marker Testing.

5.3 Clinical data collection

Extracting each patient's age, gender, pathological type, treatment plan, drug dosage and cycle, pre-treatment clinical staging, imaging results, etc., from the medical record system. The clinical efficacy is judged, and the evaluation of the objective response rate uses the RECIST 1.1 criteria.

6 SAMPLE AND STATISTICS

6.1 Non-inferiority Test Sample Size Calculation:

Two-sided alpha (α) = 0.05, Power (1-β) = 80%, 3-year Disease Progression Survival (DPS) = 75%, Non-inferiority margin = 20%, Estimated minimum sample size per group = 74

Considering a 20% dropout rate, the final planned enrollment per group is 100 cases, totaling 200 cases for both groups.

6.2 Consistency Comparison of Recurrence and Metastasis Cases:

Analyzing the Specificity and Sensitivity of the PTC Drug Sensitivity Test. Consistency analysis variables include: positive agreement rate, negative agreement rate, and overall agreement rate. The comparison of drug sensitivity test results with clinical efficacy uses Table 3 and Table 4.

Table 3: Consistency Test for Recurrence and Metastasis Cases

**Drug**	**Clinical Outcome**		**Total**
**Sensitivity Test**	CR+PR	SD+PD	
**Effective**	a	B	a+b
**Ineffective**	c	D	c+d
**Total**	a+c	b+d	T1

Calculation Method: Positive Agreement Rate = aa+c×100%*a*+*ca*×100%

Negative Agreement Rate = db+d×100%*b*+*dd*×100%

Overall Consistency Rate = a+dT1×100%*T*1*a*+*d*×100%

Sensitivity = aa+b×100%*a*+*ba*×100%

Specificity = db+d×100%*b*+*dd*×100%

Table 4: Consistency Test for Lesion Changes

Drug Sensitivity Test	Clinical Outcome		Total
	Decrease	Increase	
**Effective**	A	B	A+B
**Ineffective**	C	D	C+D
**Total**	A+C	B+D	T2

Calculation Method: Positive Agreement Rate =A/(A+C)×100%

Negative Agreement Rate =D/(B+D)×100%

Overall Consistency Rate = (A+D)/(T2)×100%

Sensitivity =A/(A+B) ×100%

Specificity =D/(B+D) ×100%

6.3 **Data Analysis Methods**

Statistical analysis is performed using SPSS 24 software. Descriptive analysis is employed for patient baseline information. Categorical data is described using frequencies and proportions (%), while continuous data is presented as mean ± standard deviation (x±s), median, maximum value, minimum value, and interquartile range.

All hypothesis tests are two-tailed, and a P-value less than 0.05 is considered to indicate statistically significant differences. Comparative evaluation of baseline data between groups is conducted using two-sided statistical tests at a significance level of α=0.05.

7 TUMOR WHOLE EXOME SEQUENCING (WES) BIOINFORMATICS DATA COLLECTION

Whole exome sequencing refers to the genomic analysis method that enriches DNA in the exon regions of the entire genome using sequence capture or target technology, followed by high-throughput sequencing. There are approximately 180,000 exons in the human genome, accounting for 1% of the genome, or about 30MB. The protein-coding regions of the human genome contain about 85% of pathogenic mutations. WES is mainly used to identify and study coding regions and UTR areas related to diseases and population evolution, as well as structural variations. By

combining WES results with a large number of public bioinformatics databases, the analysis of tumor-related molecular markers such as gene mutations, TMB, MSI, MMR, etc., can better explain the associations and pathogenic mechanisms among the obtained variation structures.

7.1 Tumor Mutational Burden (TMB)

TMB, also known as TML (Tumor Mutation Load), refers to the number of gene mutations contained in an average 1 Mb (1 million base pairs) of the tumor genome. TMB is calculated based on whole exome sequencing by determining the number of somatic mutations after removing germline mutations in the tumor genome. It can be quantitatively analyzed using the total number of non-synonymous mutations or the number of mutations per 1 Mb. A mutation load of 10 per 1 Mb is equivalent to the presence of 150 non-synonymous mutations in the genomic coding region. However, among these 150 non-synonymous mutations, only 1 to 2 neoantigens may be produced. In theory, the higher the TMB, the more neoantigens that can be recognized by T-lymphocytes, making it more likely to be a target for immunotherapy.

7.2 Mismatch Repair (MMR) and Microsatellite Instability (MSI)

Mismatch Repair (MMR) genes are highly conserved housekeeping genes that repair DNA base mismatches, ensuring high fidelity during DNA replication and maintaining genomic stability. Microsatellites (MS), also known as simple sequence repeats, are short, repetitive DNA sequences composed of 1 to 6 nucleotides that can be repeated 20 to 60 times or more. DNA methylation or gene mutations causing MMR gene defects lead to changes in the length of microsatellite repeat sequences, a phenomenon known as Microsatellite Instability (MSI). It is mainly manifested as: when a tumor cell has a deficiency in one or more mismatch repair proteins, it is called deficient Mismatch Repair (dMMR), which is characterized by high-frequency MSI (MSI-H); when there is no deficiency in mismatch repair proteins, it can be considered proficient MMR (pMMR), characterized by low-frequency MSI (MSI-L) or microsatellite stability (MSS). Therefore, MMR deficiency and MSI are associated in tumor progression. Initial studies on anti-PD-1/PD-L1 immunotherapy in colorectal cancer showed that only one patient responded, while a 2015 study found that the response rate to anti-PD-1 antibodies significantly increased in patients with dMMR colorectal cancer. Subsequent evaluations of PD-1 antibody treatment effects on 12 types of dMMR tumors also showed more clinical benefits. This suggests that although tumors originate from different sources, they are effective for the same treatment pattern due to the commonality of

dMMR at the genetic level. The reason may be that MSI induced by dMMR causes more gene mutations, releasing more neoantigens, making tumor cells more recognizable by the immune system. Blocking the PD-1/PD-L1 pathway can lift the immune suppression in the microenvironment, enhancing the immune killing effect on tumors. Previous studies have also confirmed that dMMR can activate the immune system, and dMMR colon cancer tissues are often accompanied by more lymphocyte infiltration and a rich cytokine environment. A recent retrospective study from a single center sequenced 224 colorectal cancer patients, calculated the corresponding tumor mutation burden, and simultaneously used immunohistochemistry to detect MMR-related proteins. It was found that 193 patients with pMMR had fewer than 20 mutations; among the 31 patients with 20 or more mutations, 28 cases (90%) were dMMR, which also indicates a certain connection between TMB and dMMR. Combining the two may improve predictive effects. The U.S. Food and Drug Administration (FDA) has approved Keytruda (anti-PD-1) for the treatment of all MSI-H frontline treatment-resistant advanced solid tumor patients, becoming a treatment drug that does not consider tumor type but only considers tumor markers.

7.3 Sequencing Library Construction

Genomic DNA is randomly fragmented into pieces of the length required for the capture platform. The ends of the DNA fragments are repaired, an "A" is added to the 3' end, and library adapters are ligated. The adapter-ligated library is subjected to linear amplification (LM-PCR) to prepare a hybridization library. An appropriate amount of the hybridization library is used with exon chips for capture enrichment, and unenriched fragments are washed away before amplification.

7.4 Sequencing

The amplified products are subjected to quality control (QC), and once they pass QC, they are ready for sequencing. A next-generation sequencing (NGS) platform is used for high-throughput sequencing of each qualified library, ensuring that the data volume for each sample meets the standard. The raw image data obtained from sequencing is transformed into raw sequence data (raw reads) by base-calling software, which are paired-end reads.

The data is stored in the FASTQ file format, referred to as raw data.

7.5 Data Analysis

The raw data (raw reads) may contain adapter sequences, low-quality bases, and undetermined bases (represented by "N"), which can interfere with subsequent analysis. Therefore, the raw data is first filtered to obtain clean data. Alignment software is then used to map the clean data of each sample to the human reference genome (GRCh37/HG19), resulting in BAM-formatted initial alignment files. A strict quality control system is applied throughout the analysis process to correct the quality of the alignment results. Based on the alignment results, statistical evaluations are made for each sample's sequencing depth, coverage, and alignment rate. Subsequent analysis includes the identification of genetic variations such as SNPs, InDels, SVs, CNVs, and annotation of the variation results.

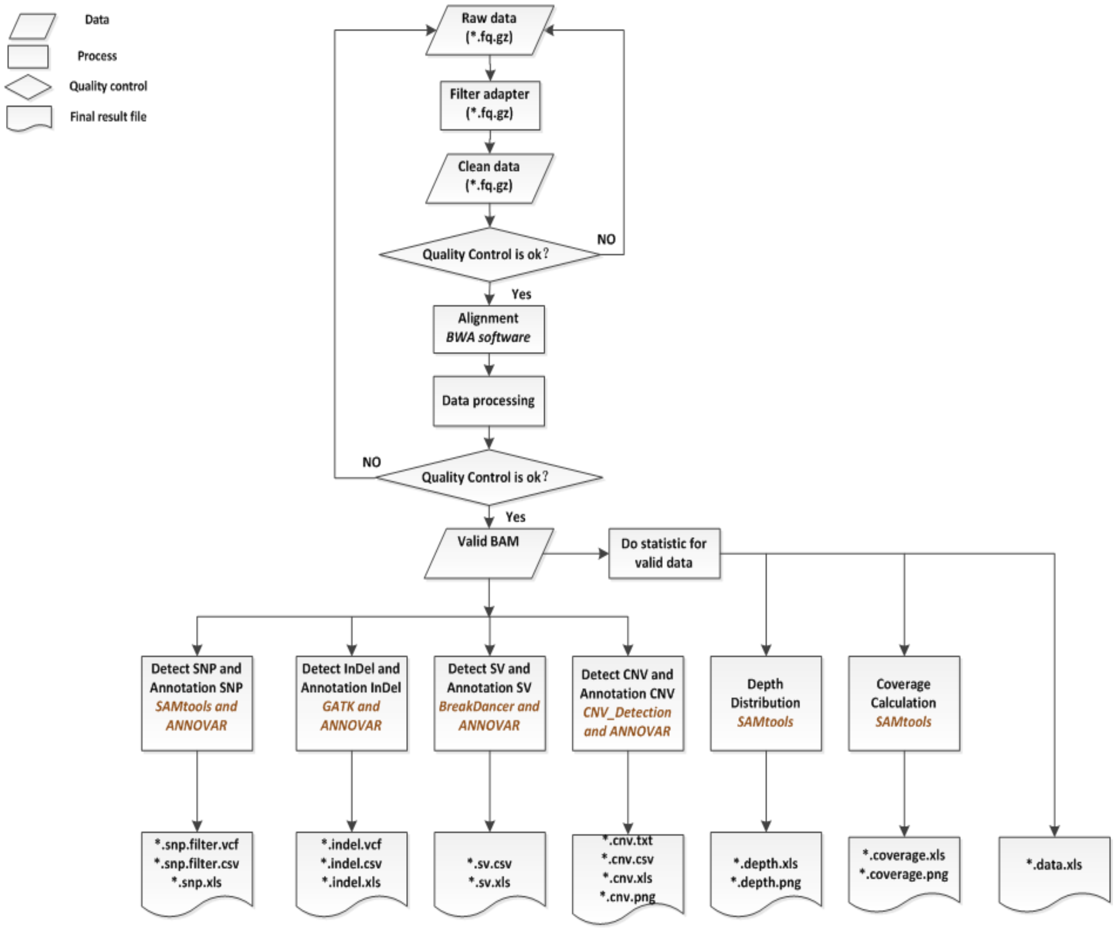

Figure 7: Data Analysis Workflow

8 QUALITY ASSURANCE AND CONTROL MEASURES

To ensure the quality of research activities meets the required standards, monitoring is conducted at every stage of data processing to ensure all data is credible and correctly handled. The sponsor is responsible for comprehensively tracking and monitoring the implementation of the clinical trial,

ensuring compliance with relevant regulatory requirements and adherence to the study protocol. Clinical trial researchers and participants understand the necessary related regulatory requirements and possess professional expertise related to clinical trials. They have the capability to design and implement relevant clinical trials, are familiar with related detection technologies, can correctly interpret test results, and have passed qualification reviews, with relatively fixed personnel. Laboratory quality control includes establishing unified laboratory standard operating procedures (SOPs) and quality control processes, conducting quality control simultaneously when performing drug sensitivity tests, and only when the quality control results are satisfactory can the data from this test be deemed valid. The person in charge of statistics has the relevant professional background and capability.

The design of case report forms (CRFs) is comprehensive, complete, and concise, facilitating easy understanding, filling out, data entry, and statistical analysis. Investigators record collected data into paper CRFs according to the study protocol requirements, which are then entered into a specifically established EDC system by trained personnel. At the end of the study, investigators will submit all CRFs for all enrolled patients to the data management center; these CRFs should be complete and signed.

9 CRITERIA FOR TERMINATING CLINICAL RESEARCH

- Serious Safety Issues: The clinical trial should be terminated promptly if serious safety issues arise during the trial process.
- Lack of Clinical Value: The trial should be discontinued if it is found that the product does not possess clinical value.
- Significant Protocol Errors: If significant flaws are found in the clinical trial protocol that make it impossible to evaluate the product's effectiveness, or if serious deviations occur during implementation that make it difficult to assess the product's effects, the trial should be terminated.
- Sponsor's Request: Termination may be requested by the sponsor for reasons such as financial constraints.
- Non-compliance with Regulations and Protocol: If the clinical trial institution and researchers do not adhere to relevant laws, regulations, and the clinical trial protocol, and do not correct the situation after being pointed out, with serious or persistent non-compliance.

- Regulatory Order: The National Medical Products Administration may order the termination of the clinical trial for certain reasons.
- Other Circumstances: Other situations that should be discussed and decided upon by the sponsor and the researcher whether to terminate the trial.

After the termination of the study, the collected data from the subjects can still be used to evaluate the safety of the product.

10 SUBJECT PROTECTION PLAN

This study adheres to the "Drug Administration Law of the People's Republic of China," the "Declaration of Helsinki" by the World Medical Association, the "Ethical Review Methods for Biomedical Research Involving Humans (Trial)," and other ethical guidelines, as well as World Health Organization guidelines related to ethical review. From the perspective of protecting the rights and safety of subjects, the clinical trial research plan is strictly reviewed. Before the start of the study, the trial protocol must be approved by the ethics committee of the medical research responsible unit before clinical trials can be implemented.

Before each patient is included in this study, the research physician has the responsibility to fully and comprehensively introduce the purpose, procedures, and potential risks of the study in written form to the patient or their designated representative. Patients should be made aware that they have the right to withdraw from the study at any time. A written informed consent form must be given to each patient before they are included in the study, and it is the responsibility of the research physician to obtain informed consent before each patient enters the study. The informed consent form should be retained as part of the clinical research documentation.

Consent must be obtained from patients before they are enrolled, authorizing the use and/or disclosure of personal and/or health data. To protect patient privacy, patient age will be recorded on the CRF without the patient's full date of birth. Experimental samples are assigned a unique number at enrollment to remove any identifying information about the subjects. Data transmitted to the sponsor, laboratories, and statistical analysts will be conducted under the study number of the subjects. This study will only collect and process clinical data that is essential for evaluating the drug sensitivity test method.

All personal information of the subjects is confidential and can only be accessed by the relevant researchers, the sponsor of this research project, members of the ethics committee, and relevant

personnel of the national/local drug regulatory authority. Researchers and sponsors are required to keep the personal information of the subjects confidential.

Reference

[1] SIEGEL R, DESANTIS C, VIRGO K, et al. Cancer treatment and survivorship statistics, 2012 [J]. CA Cancer J Clin, 2012, 62(4): 220-41.

[2] DE GRAMONT A, FIGER A, SEYMOUR M, et al. Leucovorin and fluorouracil with or without oxaliplatin as first-line treatment in advanced colorectal cancer [J]. J Clin Oncol, 2000, 18(16): 2938-47.

[3] GOLDBERG R M, SARGENT D J, MORTON R F, et al. A randomized controlled trial of fluorouracil plus leucovorin, irinotecan, and oxaliplatin combinations in patients with previously untreated metastatic colorectal cancer [J]. J Clin Oncol, 2004, 22(1): 23-30.

[4] CASSIDY J, CLARKE S, DIAZ-RUBIO E, et al. Randomized phase III study of capecitabine plus oxaliplatin compared with fluorouracil/folinic acid plus oxaliplatin as first-line therapy for metastatic colorectal cancer [J]. J Clin Oncol, 2008, 26(12): 2006-12.

[5] YIN S, XI R, WU A, et al. Patient-derived tumor-like cell clusters for drug testing in cancer therapy [J]. Sci Transl Med, 2020, 12(549).

[6] SEVIN B U, PENG Z L, PERRAS J P, et al. Application of an ATP-bioluminescence assay in human tumor chemosensitivity testing [J]. Gynecol Oncol, 1988, 31(1): 191-204.

[7] GAO H, KORN J M, FERRETTI S, et al. High-throughput screening using patient-derived tumor xenografts to predict clinical trial drug response [J]. Nat Med, 2015, 21(11): 1318-25.
