# Supplementary material for: In vitro 3D drug sensitivity testing for patient-derived tumor-like cell clusters and whole-exome sequencing to personalize postoperative treatment: A study protocol for a multicenter randomized controlled trial
Source: PLoS One. 2025 Jul 14;20(7):e0326760. doi: 10.1371/journal.pone.0326760 (PMC12258565; doi:10.1371/journal.pone.0326760)
Supplement: Attachment — Submitted filename: Response to Reviewers.docx [file pone.0326760.s003.docx]

**Response to Reviewers**

**Journal Requirements:**

**1. Please ensure that your manuscript meets PLOS ONE's style requirements, including those for file naming. The PLOS ONE style templates can be found at https://journals.plos.org/plosone/s/file?id=wjVg/PLOSOne_formatting_sample_main_body.pdf and** [**https://journals.plos.org/plosone/s/file?id=ba62/PLOSOne_formatting_sample_title_authors_affiliations.pdf**](https://journals.plos.org/plosone/s/file?id=ba62/PLOSOne_formatting_sample_title_authors_affiliations.pdf)

Thank you for the reminder. We have made the format adjustments in accordance with the website examples.

**2. Thank you for submitting your clinical trial to PLOS ONE and for providing the name of the registry and the registration number. The information in the registry entry suggests that your trial was registered after patient recruitment began. PLOS ONE strongly encourages authors to register all trials before recruiting the first participant in a study.**

**As per the journal’s editorial policy, please include in the Methods section of your paper: 1) your reasons for your delay in registering this study (after enrolment of participants started); 2) confirmation that all related trials are registered by stating: “The authors confirm that all ongoing and related trials for this drug/intervention are registered.**

We are well aware that prospective trial registration is the best practice for publication. However, in this study, the registration occurred after the commencement of patient recruitment due to the following reasons: As the Chinese Clinical Trial Registry still allowed delayed registration after study began on 2021, we initiated recruitment after obtaining ethical approval. Sometime later, we realized that ClinicalTrials encourages authors to register before recruitment. Upon realizing the delay in registration, we immediately completed the trial registration on ClinicalTrials and ensured that all relevant information is accurate and complete.

In the "Methods" section of the manuscript, we have provided a detailed explanation of the reasons for the delayed registration and confirmed that all related clinical trials have been registered. And we commit that all future related trials will strictly adhere to the requirements of prospective registration.

We understand the importance of timely registration for the transparency, ethics, and credibility of research. We hope that through the above remedial actions and commitments, we can ensure the integrity and credibility of this study and meet the publication standards of PLOS ONE.

**3. Thank you for stating the following financial disclosure: “Guole Lin National High Level Hospital Clinical Research Funding (No. 2022-PUMCH-B-005)” Please state what role the funders took in the study.  If the funders had no role, please state: "The funders had no role in study design, data collection and analysis, decision to publish, or preparation of the manuscript." If this statement is not correct you must amend it as needed. Please include this** **amended Role of Funder statement in your cover letter; we will change the online submission form on your behalf.**

The funders had no role in study design, data collection and analysis, decision to publish, or preparation of the manuscript. And we have included amended Role of Funder statement in cover letter.

**4. Please note that funding information should not appear in the Acknowledgments section or other areas of your manuscript. We will only publish funding information present in the Funding Statement section of the online submission form. Please remove any funding-related text from the manuscript.**

We have removed funding-related text from the manuscript.

**5. Your ethics statement should only appear in the Methods section of your manuscript. If your ethics statement is written in any section besides the Methods, please delete it from any other section.**

We have removed ethics statement from manuscript except Methods section.

**6. Please remove your figures from within your manuscript file, leaving only the individual TIFF/EPS image files, uploaded separately. These will be automatically included in the reviewers’ PDF.**

We have removed figures from manuscript.

**7. Please include captions for your Supporting Information files at the end of your manuscript, and update any in-text citations to match accordingly. Please see our Supporting Information guidelines for more information: http://journals.plos.org/plosone/s/supporting-information.**

We have included captions for Supporting Information files at the end of the manuscript.

**8.** **We note that the original protocol file you uploaded contains a confidentiality notice indicating that the protocol may not be shared publicly or be published. Please note, however, that the PLOS Editorial Policy requires that the original protocol be published alongside your manuscript in the event of acceptance. Please note that should your paper be accepted, all content including the protocol will be published under the Creative Commons Attribution (CC BY) 4.0 license, which means that it will be freely available online, and any third party is permitted to access, download, copy, distribute, and use these materials in any way, even commercially, with proper attribution.**

**Therefore, we ask that you please seek permission from the study sponsor or body imposing the restriction on sharing this document to publish this protocol under CC BY 4.0 if your work is accepted. We kindly ask that you upload a formal statement signed by an institutional representative clarifying whether you will be able to comply with this policy. Additionally, please upload a clean copy of the protocol with the confidentiality notice (and any copyrighted institutional logos or signatures) removed.**

We apologize for any confusion caused and comply with the publication requirements for open access to our research protocol. This article is licensed under a Creative Commons Attribution 4.0 International License. We have updated the protocol and upload a formal statement as Supporting Information file.

**9. Please review your reference list to ensure that it is complete and correct. If you have cited papers that have been retracted, please include the rationale for doing so in the manuscript text, or remove these references and replace them with relevant current references. Any changes to the reference list should be mentioned in the rebuttal letter that accompanies your revised manuscript. If you need to cite a retracted article, indicate the article’s retracted status in the References list and also include a citation and full reference for the retraction notice.**

Thank you for the reminder. We have carefully reviewed our references and confirm there are no citations of retracted articles. All listed references are current and valid.

**Reviewer #1:**

**1. Section 2.7.3 is the start time for the time to event endpoints the date of randomisation? If date of surgery there may be a bias, as patients that are not randomised after surgery will be excluded from the time to event curves. Please state the start time for the endpoints in the article.**

Thank you for your review and question. In this study, the start time for the time to event endpoints is defined from the surgical date to the endpoint. Given the specific inclusion criteria, pathological staging is essential to determine the necessity of adjuvant chemotherapy for patients. The randomization process commences once the pathological results are available (after surgery). Patients who are not randomized post-surgery are considered ineligible per the study's inclusion criteria and, therefore, should be excluded from the analysis. Thus, different from other clinical trials, using the surgical date as the event's starting point will not introduce bias into our study.

**2. Will Kaplan Meier curves be developed by randomisation arm for DFS?
Will log rank tests be used if there is any censoring to test for differences in the primary endpoint by arm? Please add to methods.**

Thank you for your reminder, and we initially missed certain elements pertaining to survival analysis. We have now addressed by incorporating further details into our manuscript. We plan to use Kaplan-Meier curves for DFS, and use the Log-rank test to compare the primary endpoint between different arms in the presence of censoring.
